# 5/6 Nephrectomy as an Experimental Model for Chronic Kidney Disease: New Vasoactive and Antioxidant Therapeutic Targets

**DOI:** 10.3390/ph19050676

**Published:** 2026-04-26

**Authors:** Regina Souza Aires, Maria da Conceição Correia Silva, Filipe de Melo Barbosa, Mirelly Cunha da Silva, Silvia Maria de Luna Alves, Alice Valença Araújo, Thyago Moreira de Queiroz

**Affiliations:** Centro Acadêmico de Vitória, Universidade Federal de Pernambuco (UFPE), Vitória de Santo Antão 55608-680, PE, Brazil; regina.souzaa@ufpe.br (R.S.A.); maria.mccs@ufpe.br (M.d.C.C.S.); filipe.melobarbosa@ufpe.br (F.d.M.B.); mirelly.cunha@ufpe.br (M.C.d.S.); silvia.lunaalves@ufpe.br (S.M.d.L.A.); alice.araujo@ufpe.br (A.V.A.)

**Keywords:** cardiorenal syndrome, endothelial dysfunction, oxidative stress, inflammatory signaling, renin–angiotensin system

## Abstract

Chronic kidney disease (CKD) is a progressive disorder characterized by declining renal function and increased cardiovascular risk. Experimental models are essential for investigating these mechanisms, and the 5/6 nephrectomy (5/6 Nx) model is widely used to reproduce cardiorenal alterations observed in CKD. This review aims to critically evaluate how effectively the 5/6 Nx model reproduces vasoactive and redox mechanisms relevant for pharmacological testing. A narrative synthesis of experimental studies using the 5/6 Nx model in rodents was performed, focusing on vascular, inflammatory, and oxidative pathways. The 5/6 Nx model reproduces major CKD features, including hypertension, proteinuria, glomerulosclerosis, and cardiovascular remodeling. Early activation of the renin–angiotensin–aldosterone system, endothelin signaling, and sympathetic pathways contributes to vascular dysfunction. Sustained oxidative stress reduces nitric oxide bioavailability and promotes endothelial dysfunction. Dysregulation of natriuretic peptides and increased 20-HETE signaling further contribute to vascular imbalance and remodeling. These alterations occur in a well-defined temporal progression, supporting the use of this model for mechanistic and pharmacological studies. The 5/6 Nx model remains a robust and translationally informative platform for investigating CKD progression, provided that pathway-specific reproducibility and experimental variables are carefully considered.

## 1. Introduction

Chronic kidney disease (CKD) is a major global health condition, defined by persistently reduced glomerular filtration rate (GFR) or elevated albuminuria for more than three months [1]. Epidemiological data indicate that CKD affects a substantial proportion of the global population and remains a leading cause of mortality, accounting for over one million deaths worldwide in recent estimates [1,2]. Although therapeutic approaches have advanced, disease progression from early renal impairment to end-stage kidney disease continues to impose significant clinical and economic burdens. Healthcare expenditures increase with disease progression, while quality of life declines.

In Brazil, CKD is an increasingly relevant public health concern. National survey-based estimates suggest a self-reported prevalence of 1.4% among adults [3], whereas laboratory-based analyses report higher prevalence values, reaching approximately 3.8% among insured populations [4]. Furthermore, in individuals with hypertension or diabetes, two of the most significant risk factors, previously unrecognized CKD has been identified in up to 15.4%, highlighting substantial underdiagnosis in high-risk groups [5]. These findings underscore ongoing challenges related to early detection, diagnosis, and equitable access to nephrology evaluation across the Brazilian healthcare system.

Despite advances in therapeutic strategies, including renin–angiotensin–aldosterone system (RAAS) inhibitors, sodium–glucose cotransporter-2 (SGLT2) inhibitors, and mineralocorticoid receptor antagonists, current treatments primarily slow CKD progression rather than prevent or reverse renal damage [1]. This limitation reflects the complex nature of CKD pathophysiology, which extends beyond renal impairment to include systemic cardiovascular dysfunction. Declining renal function promotes neurohormonal activation, particularly of the RAAS, along with chronic inflammation, oxidative stress, and endothelial dysfunction, key drivers of hypertension and vascular injury [6,7]. In addition, the accumulation of uremic toxins enhances endothelial activation and pro-inflammatory signaling via NF-κB and reactive oxygen species (ROS) pathways [8,9], resulting in reduced nitric oxide (NO) bioavailability, increased vasoconstrictor, and cardiovascular risk [10].

Although clinical studies are essential for establishing epidemiological associations and evaluating therapeutic interventions, they face inherent limitations in mechanistic investigation due to population heterogeneity, comorbidities, and ethical constraints [11]. Controlled mechanistic studies using experimental models help overcome these barriers by enabling precise manipulation of specific pathways, longitudinal tissue sampling, and integration of molecular, hemodynamic, and structural data. Preclinical validation remains indispensable for identifying therapeutic targets, such as vasoactive mediators or redox-modulating systems, and for ensuring the safety and efficacy of candidate interventions prior to translation into human trials [12].

Among available experimental models, the 5/6 nephrectomy (5/6 Nx) model is one of the most established and extensively characterized platforms for studying CKD pathophysiology. Originally developed to mimic progressive renal mass loss [13], this model reproduces key features of human CKD, including hypertension, proteinuria, glomerulosclerosis [14,15,16], inflammation [17], and cardiac remodeling [18]. By inducing a controlled reduction in renal mass, 5/6 Nx promotes predictable decline in renal function, activation of the RAAS, and systemic vascular alterations [19], providing a robust and reproducible model for investigating the temporal progression of CKD.

This review provides an updated overview of 5/6 Nx as an experimental model to study CKD progression. It synthesizes the temporal evolution of vascular, inflammatory, and redox alterations, highlights emerging vasoactive and antioxidant therapeutic targets, and evaluates how effectively the model reproduces key vasoactive and oxidative pathways relevant to pharmacological testing and translational application.

## 2. The 5/6 Nephrectomy Model of CKD

### 2.1. Historical Development of the Model

The 5/6 nephrectomy (5/6 Nx), also known as the remnant kidney model, represents one of the most widely used experimental strategies to reproduce progressive CKD in rodents. The conceptual framework of this model dates back to the early twentieth century, when Chanutin et al., 1932 [13] first demonstrated that subtotal renal ablation leads to chronic uremia and progressive renal structural damage, establishing the principle that loss of nephron mass can lead to the development of chronic renal failure. This seminal work laid the foundation for experimental models of CKD by showing that abrupt reduction in renal mass produces long-term functional and morphological alterations in the remaining kidney tissue [13].

Early experimental evidence of renal insufficiency induced by nephron loss was provided by Platt et al., 1952 [20], who demonstrated that reduction in renal function leads to significant alterations in renal hemodynamics and nitrogen metabolism. Building on these observations, studies in the 1960s established the remnant kidney model in rodents as a reproducible platform to investigate chronic renal insufficiency. In this context, Morrison (1962) [14] showed that surgical reduction of renal mass in rats results in progressive renal dysfunction and structural damage, laying the foundation for the development of the 5/6 Nx model. Subsequently, investigations in the 1960s and 1970s further refined this model and advanced the understanding of the mechanisms underlying renal injury after nephron loss. Studies conducted in large animal models, particularly dogs, demonstrated that a marked reduction in nephron number induces compensatory increases in single-nephron filtration rate and renal blood flow, establishing the concept of adaptive hyperfiltration as a central mechanism driving progressive renal injury [21].

A landmark study by Shimamura and Morrison (1975) [22] provided one of the first detailed descriptions of the structural evolution of renal injury following subtotal nephrectomy. Using rat models of renal ablation, the authors demonstrated progressive glomerular hypertrophy, mesangial expansion, and eventual glomerulosclerosis in the remnant kidney. These findings established the 5/6 Nx as a reliable experimental system for studying progressive renal injury and its underlying mechanisms [22].

Further mechanistic insight emerged in the early 1980s with the work of Hostetter and colleagues [23], who proposed that compensatory glomerular hyperfiltration and intraglomerular hypertension in the remaining nephrons represent key drivers of progressive renal injury following nephron loss. Their studies demonstrated that increased glomerular capillary pressure and single-nephron filtration rate contribute directly to structural damage and progressive sclerosis of the glomeruli [23]. This concept of maladaptive hyperfiltration remains one of the central paradigms in CKD pathophysiology. Subsequent investigations confirmed that reduction of renal mass triggers a cascade of maladaptive responses involving multiple pathophysiological pathways. The model has been extensively validated and reproduces major features of human CKD, including vascular dysfunction and systemic complications [19,24,25].

Over the past decades, methodological refinements have established the 5/6 Nx as a standardized and widely used platform for investigating CKD pathophysiology. The model has been extensively applied to study mechanisms involved in renal fibrosis, endothelial dysfunction, oxidative stress, and cardiorenal interactions, as well as to evaluate potential therapeutic strategies targeting these pathways. Importantly, the 5/6 Nx reproduces several hallmark features of progressive human CKD, including hypertension, proteinuria, glomerulosclerosis, tubulointerstitial fibrosis, and cardiovascular remodeling, and exhibits a predictable temporal progression of renal and vascular injury, making it a valuable tool for mechanistic and preclinical studies [19,25,26].

### 2.2. Surgical Approaches: One-Step vs. Two-Step Nephrectomy

Subtotal nephrectomy in the 5/6 Nx model is achieved by reducing approximately five-sixths of total renal mass through complete removal of one kidney combined with partial ablation of the contralateral kidney. Two main surgical techniques are employed: parenchymal resection (pole excision), originally described in early experimental studies [13,14], and infarction-based ablation, performed by ligation of segmental renal arteries [14,27]. In the resection approach, the upper and lower poles are surgically excised, allowing for controlled removal of renal parenchyma, whereas the infarction method induces localized ischemic necrosis through selective arterial ligation. Although both techniques achieve a comparable reduction in nephron number, they differ mechanistically in the extent of ischemic injury, inflammatory activation, and neurohumoral responses, which may influence disease severity and vascular outcomes [28,29].

**Figure 1:**
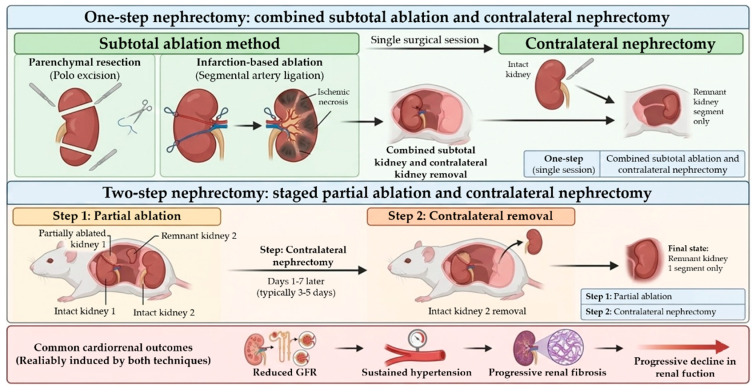
Schematic overview of the rodent 5/6 nephrectomy (5/6 Nx), depicting the stages and single-step surgical strategies employed to achieve subtotal renal mass reduction. Experimental approaches to the 5/6 nephrectomy (5/6 Nx) model and associated cardiorenal outcomes. The upper panel illustrates the one-step procedure, in which subtotal renal mass reduction (via parenchymal excision or infarction induced by segmental artery ligation) is combined with contralateral nephrectomy in a single surgical session. The middle panel depicts the two-step procedure, consisting of initial partial ablation of one kidney followed by contralateral nephrectomy after a short interval (typically 3–7 days). Both approaches result in a marked reduction of functional renal mass, leading to compensatory hyperfiltration, activation of vasoactive systems, and increased oxidative stress. These alterations contribute to the development of reduced GFR, sustained hypertension, progressive renal fibrosis, and a gradual decline in renal function, recapitulating key features of CKD. (Image generated using Google Gemini: https://gemini.google.com and edited by the authors using Microsoft PowerPoint—Microsoft 365, 2019).

In addition to the surgical technique, 5/6 Nx can be performed using two-step or one-step protocols (Figure 1). The two-step approach, widely used in rat models, consists of partial ablation of one kidney followed by contralateral nephrectomy after a recovery period, resulting in improved perioperative survival and greater hemodynamic stability [28,30]. In contrast, the one-step procedure combines both interventions in a single surgery, offering technical simplicity and shorter experimental timelines but potentially inducing a more pronounced acute inflammatory response and higher early mortality, depending on species and experimental conditions. Despite these procedural differences, both approaches consistently reproduce the hallmark features of progressive CKD, including decline in GFR, sustained hypertension, and progressive renal fibrosis [31].

The reproducibility and well-characterized temporal progression of the 5/6 Nx model make it particularly suitable for evaluating mechanistic pathways and testing pharmacological interventions. However, its translational relevance depends on how closely these experimental conditions reflect human CKD.

### 2.3. Strengths of the Model in Translational Research

The 5/6 Nx offers several advantages that make it particularly valuable for studying CKD mechanisms. One of the major strengths of this model is its ability to reproduce a predictable and progressive trajectory of CKD, including hypertension, albuminuria, glomerulosclerosis, tubulointerstitial fibrosis, and cardiovascular remodeling [22,24,26]. These features allow for simultaneous investigation of renal injury, vascular dysfunction, and systemic complications. In addition, the model enables longitudinal analysis of molecular and hemodynamic alterations, facilitating the identification of therapeutic targets within pathways such as the RAAS, endothelin signaling, oxidative stress, and inflammatory cascades.

Because these pathways are central to current and emerging pharmacological strategies, the 5/6 Nx model is particularly valuable for preclinical testing. It allows for the evaluation of drug effects on both renal and cardiovascular endpoints, providing a comprehensive assessment of therapeutic potential.

### 2.4. Limitations and Experimental Variables

Despite its reproducibility, the phenotype induced by the 5/6 Nx model varies according to experimental conditions. Surgical technique (resection versus infarction), species and strain, sex, age, and dietary factors significantly influence the severity and progression of renal injury [26,32,33].

Genetic background critically modulates susceptibility to renal injury and the progression of CKD in experimental models. In mice, C57BL/6 strains exhibit relative resistance, whereas 129/Sv strains develop more severe glomerulosclerosis and interstitial fibrosis, highlighting the impact of intrinsic genetic determinants on disease severity [16,33,34]. In rats, strain-dependent differences are also evident in the 5/6 Nx model: Sprague-Dawley rats are more susceptible to CKD progression, whereas Wistar rats display relative resistance, a phenotype associated with enhanced intrarenal NO signaling, including increased inducible nitric oxide synthase (iNOS) expression in the cortex and endothelial nitric oxide synthase (eNOS) in the medulla [35]. Additional comparative studies further demonstrate that Wistar rats may develop a more severe hypertensive and proteinuric phenotype compared with inbred strains such as Lewis et al. (2025) [11], which exhibit attenuated renal injury, reduced inflammatory activation, and slower functional decline. These differences are mechanistically linked to strain-specific variations in nephron endowment, immune responsiveness, and vasoactive pathway regulation, ultimately determining the balance between adaptive and maladaptive responses following nephron loss [36].

Sex hormones play an important role in modulating CKD progression. Experimental studies demonstrate that female animals generally exhibit slower disease progression and reduced oxidative stress compared with males. Estrogen signaling appears to exert protective effects by attenuating inflammatory activation and improving antioxidant defenses [37]. For instance, estrogen replacement has been shown to mitigate multiorgan oxidative injury in female rats subjected to chronic renal failure induced by 5/6 Nx [38]. These findings highlight the importance of considering sex as a biological variable in experimental CKD research.

Age is another critical determinant of disease progression. Older animals exhibit reduced nephron reserve, impaired regenerative capacity, and heightened susceptibility to fibrosis, which may accelerate the decline in renal function following nephron loss. In experimental studies, aged animals subjected to 5/6 Nx develop more pronounced hypertension, cardiac remodeling, and glomerular injury compared with younger counterparts [33]. These observations parallel clinical findings in humans, where aging is a major risk factor for CKD progression.

Dietary composition represents a major modifier of the CKD phenotype in the 5/6 Nx. High-salt diets can exacerbate hypertension and vascular dysfunction by enhancing RAAS activation and impairing endothelial function [39]. Similarly, high-phosphate diets accelerate cardiovascular complications, including vascular calcification and arterial stiffness, thereby improving the translational relevance of the model to mineral–bone disorders associated with advanced CKD [30,40]. High-protein diets may further aggravate renal injury by increasing glomerular hyperfiltration and metabolic workload in the remaining nephrons [15].

Moreover, certain complications of advanced CKD, such as vascular calcification and mineral bone disorders, may not fully develop without additional metabolic stressors (e.g., high-phosphate diets). These variables, together with the absence of common comorbidities such as diabetes, should be considered when interpreting results and may limit the direct extrapolation of findings to human CKD.

## 3. Vasoactive Pathways Driving Cardiorenal Dysfunction in 5/6 Nx

The progression of CKD in the 5/6 Nx model is driven by the early and sustained activation of interconnected vasoactive systems that regulate vascular tone, renal hemodynamics, and extracellular volume. These pathways operate as an integrated network in which neurohumoral activation, oxidative stress, and endothelial dysfunction reinforce one another, creating a self-amplifying cycle of vascular injury. The ability of the 5/6 Nx model to reproduce these mechanisms supports its value as a controlled experimental platform for investigating cardiorenal dysfunction and evaluating pharmacological interventions.

### 3.1. Renin–Angiotensin–Aldosterone System (RAAS)

Activation of the RAAS is one of the earliest and most consistently observed alterations in the 5/6 Nx model and plays a central role in the progression of cardiorenal dysfunction [41]. Reduction in renal mass stimulates renin release from juxtaglomerular cells, initiating a cascade in which angiotensinogen is converted to angiotensin I (Ang I) and subsequently to angiotensin II (Ang II) by angiotensin-converting enzyme (ACE) [41,42,43].

**Figure 2:**
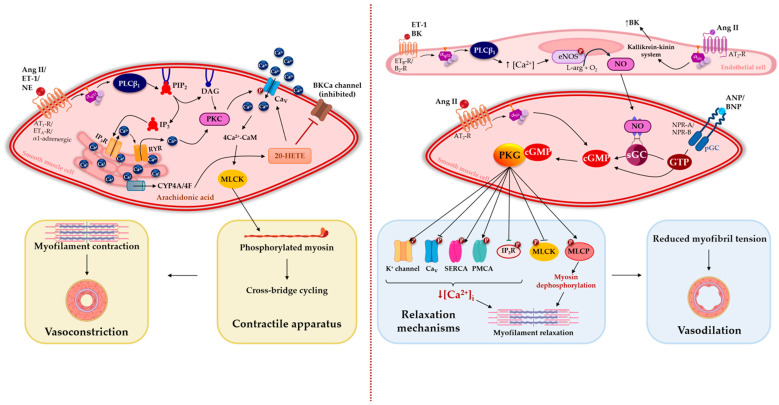
Integrated signaling pathways regulating vascular smooth muscle tone. The diagram illustrates the balance between contractile and relaxant stimuli in vascular smooth muscle cells. **Left panel** (vasoconstriction): Activation of G_q_-coupled receptors (AT_1_-R, ET_A_-R, and α1-adrenergic receptors) by angiotensin II (Ang II), endothelin-1 (ET-1), and norepinephrine (NE), as well as the 20-HETE pathway (via CYP4A/4F), stimulates phospholipase C (PLCβ_1_), leading to the generation of inositol 1,4,5-triphosphate (IP_3_) and diacylglycerol (DAG). This results in Ca^2+^ release from the sarcoplasmic reticulum and protein kinase C (PKC) activation, increasing intracellular Ca^2+^ levels and activating myosin light chain kinase (MLCK), thereby promoting contraction. Ryanodine receptor (Ryr); Large-conductance Ca^2+^-activated K^+^ channel (BKCa). **Right panel** (vasodilation): Vasorelaxation is mediated by nitric oxide (NO), bradykinin (BK), AT_2_ receptor signaling, and natriuretic peptides (ANP/BNP). AT_2_ receptor activation may enhance local BK generation, which subsequently stimulates endothelial B2 receptors, leading to NO release. NO activates soluble guanylate cyclase (sGC), whereas ANP/BNP bind to natriuretic peptide receptors (NPRs), both increasing cyclic guanosine monophosphate (cGMP) levels. cGMP activates protein kinase G (PKG), which promotes phosphorylation of many proteins, including Ca^2+^ sequestration via SERCA, extrusion via PMCA, and activation of myosin light chain phosphatase (MLCP), resulting in vascular relaxation. (Created with BioRender: https://biorender.com/ created by Regina Souza Aires in 5 March 2026 and modified using Microsoft PowerPoint).

In this model, Ang II acts as a key mediator of renal and vascular injury by promoting efferent arteriolar vasoconstriction, increasing intraglomerular pressure, and contributing to proteinuria, hypertension, endothelial dysfunction, and renal hypoxia [44,45]. These effects are primarily mediated by AT_1_ receptor activation, which triggers G_q/11_-dependent signaling pathways leading to increased intracellular calcium and vascular smooth muscle contraction, while AT_2_ receptors exert counter-regulatory vasodilatory effects [46,47,48,49] (Figure 2).

Studies evaluating the angiotensin pathway in isolated aortic vessels from rats subjected 5/6 Nx remain scarce, but an increase has been observed in the expression of AT_1_ receptors, myocardial hypertrophy and elevated serum levels of vasoconstrictor substances and ACE activity in rats, as well as endothelial dysfunction and increased levels of pro-inflammatory cytokines, accelerating the atherogenesis process via the RAAS [33,39,50,51,52,53].

In addition, aldosterone acts as a downstream effector, promoting sodium retention, inflammation, oxidative stress, and fibrosis through mineralocorticoid receptor activation [44,50].

Pharmacological blockade of RAAS components, one of the current treatments for CKD, attenuates hypertension, reduces proteinuria, and limits renal damage, highlighting the translational relevance of the 5/6 Nx model for evaluating RAAS-targeted therapies.

### 3.2. Endothelin System

The endothelin (ET) system is a key vasoactive pathway contributing to vascular dysfunction in the 5/6 Nx model. Endothelin-1 (ET-1), the predominant vascular isoform, is a potent vasoconstrictor peptide generated from its precursor through endothelin-converting enzyme (ECE) [54,55]. Its biological effects are mediated by ET_A_ and ET_B_ receptors. Activation of ET_A_ receptors in vascular smooth muscle cells promotes vasoconstriction via G_q/11_-dependent signaling and increased intracellular calcium, whereas activation of ET_B_ receptors exert dual effects: they contribute to vasoconstriction in smooth muscle cells and induce NO release and vasodilation when expressed in the endothelium [46,54,56] (Figure 2).

In the 5/6 Nx model, this balance is shifted toward vasoconstriction, favoring endothelial dysfunction and increased vascular resistance. Experimental evidence demonstrates elevated ET-1 levels in vascular tissues, including the thoracic aorta and mesenteric arterial bed, indicating upregulation of this pathway during CKD progression [57,58]. This increase contributes to hypertension and impaired vascular function.

The endothelin system also interacts closely with other vasoactive pathways. For example, Ang II stimulates ET-1 production, while ET-1 enhances Ang II–induced vasoconstriction, establishing a synergistic mechanism that amplifies vascular injury [59,60,61]. This cross-talk is particularly relevant in the 5/6 Nx model, where concurrent activation of neurohumoral systems drives disease progression.

### 3.3. Sympathetic Nervous System (SNS)

Activation of the SNS is a key neurohumoral mechanism that contributes to CKD progression and is consistently reproduced in the 5/6 Nx model. Reduction in renal mass enhances afferent signaling from the injured kidney to central autonomic nuclei, leading to sustained sympathetic overactivity [62,63].

In this model, increased sympathetic drive promotes vasoconstriction through activation of α1-adrenergic receptors in vascular smooth muscle cells, triggering G_q/11_-mediated signaling pathways that increase intracellular calcium and vascular tone (Figure 2). These alterations contribute to systemic hypertension, reduced renal blood flow, impaired sodium excretion, and progressive renal injury [62,63,64,65,66,67,68,69]. Experimental evidence further demonstrates increased norepinephrine (NE) levels and enhanced activity in central autonomic regions, such as the posterior hypothalamus and locus coeruleus, within weeks after 5/6 Nx [66].

Disruption of renal afferent signaling, through renal denervation or dorsal rhizotomy, attenuates sympathetic activation and reduces blood pressure, proteinuria, and glomerulosclerosis, highlighting the critical role of renal sensory nerves in disease progression [63,65]. In addition, the SNS interacts closely with other vasoactive systems, particularly the RAAS, forming a feed-forward mechanism that amplifies vasoconstriction, oxidative stress, and inflammation [67,69].

Therapeutic strategies targeting sympathetic activity have shown beneficial effects in experimental models. Pharmacological interventions, including β-adrenergic blockade and RAAS inhibition, reduce hypertension and renal injury, while combined approaches, such as angiotensin receptor blockade with renal denervation, produce additive effects, reinforcing the translational relevance of neurohumoral modulation in CKD [68,69,70].

### 3.4. Arachidonic Acid Metabolites

Metabolites of arachidonic acid generated via the cytochrome P450 (CYP) pathway, particularly 20-hydroxyeicosatetraenoic acid (20-HETE), represent an important vasoactive system involved in the regulation of vascular tone and CKD progression in the 5/6 Nx model. In this context, increased expression of CYP4A/4F enzymes leads to elevated 20-HETE production in both the renal microcirculation and systemic vasculature [71,72]. This eicosanoid exerts potent vasoconstrictor effects by inhibiting potassium channels and enhancing calcium influx in vascular smooth muscle cells, resulting in increased vascular resistance and hypertension [72] (Figure 2).

In the 5/6 Nx model, elevated 20-HETE levels are associated with endothelial dysfunction, oxidative stress, and vascular remodeling. Mechanistically, 20-HETE acts as a downstream mediator of other vasoactive systems, including angiotensin II and endothelin signaling, amplifying their vasoconstrictor and pro-inflammatory effects [73,74,75]. Consistent with this role, dysregulation of CYP-derived eicosanoids has been associated with accelerated renal function decline, suggesting their potential as biomarkers of disease progression [74].

Moreover, increased 20-HETE production contributes to elevated peripheral vascular resistance and promotes inflammatory and oxidative pathways that drive cardiorenal remodeling in this model [71,72]. Clinical evidence further supports the relevance of this pathway, as modulation of 20-HETE levels, for example through n-3 polyunsaturated fatty acid supplementation, has been associated with reduced blood pressure in patients with CKD, highlighting its potential as a therapeutic target [76].

### 3.5. Compensatory Failure of the Natriuretic Peptides System

In addition to vasoconstrictor pathways such as the RAAS, endothelin system, and sympathetic activation, the natriuretic peptide (NP) system acts as an important counter-regulatory mechanism in cardiovascular and renal homeostasis. Atrial natriuretic peptide (ANP) and brain natriuretic peptide (BNP), released in response to volume overload and cardiac wall stretch, promote natriuresis, diuresis, and vasodilation through activation of guanylyl cyclase-coupled receptors and subsequent cyclic guanosine monophosphate (cGMP) production [77,78,79,80,81]. In vascular smooth muscle cells, cGMP activates protein kinase G (PKG), leading to reduced intracellular Ca^2+^ levels and vasorelaxation (Figure 2).

Despite compensatory activation, the protective effects of this system are progressively impaired during CKD. Although circulating ANP and BNP levels are elevated, their biological activity is attenuated due to receptor desensitization, impaired downstream signaling, and increased degradation by neprilysin [82,83,84]. This dysfunction contributes to an imbalance favoring vasoconstriction, sodium retention, and increased glomerular pressure.

Restoration of the NP-cGMP axis has emerged as a potential therapeutic strategy. Pharmacological approaches that enhance natriuretic peptide signaling or inhibit neprilysin improve vascular function, reduce neurohumoral activation, and promote natriuresis [85,86]. In this context, angiotensin receptor–neprilysin inhibitors (ARNi) may help restore vascular homeostasis and counteract RAAS overactivation.

Overall, these pathways operate as an integrated network in which vasoconstrictor and vasodilator mechanisms interact with oxidative and inflammatory processes. In the 5/6 Nx model, this imbalance closely resembles human CKD, reinforcing its relevance for investigating vasoactive mechanisms and evaluating therapeutic strategies.

## 4. Pathophysiological Mechanisms in the 5/6 Nx Model

The 5/6 Nx model reproduces key inflammatory and oxidative mechanisms observed in human CKD, enabling the investigation of their temporal progression. Disease progression is driven by the interaction of inflammatory, oxidative, and structural processes that promote renal and vascular injury, ultimately contributing to both local tissue damage and systemic cardiovascular complications.

### 4.1. Inflammatory Signaling Pathways

The kidney contains diverse populations of resident immune cells that contribute to tissue surveillance and maintenance of renal homeostasis. During CKD progression, however, these regulatory mechanisms become dysregulated, leading to persistent inflammation and progressive fibrotic remodeling of renal tissue [87]. The 5/6 Nx model reproduces many of the inflammatory mechanisms observed in human CKD, including activation of innate immune pathways triggered by the reduction in functional renal mass [37].

Inflammation is now recognized as a major driver of CKD progression. Recruitment of innate immune cells, particularly monocytes and macrophages, together with increased expression of pro-inflammatory cytokines, chemokines, adhesion molecules, and growth factors constitutes a central pathological feature of the disease [88]. This inflammatory microenvironment disrupts renal homeostasis by impairing glomerular filtration, renal perfusion, and tubular transport processes. Sustained injury to podocytes, tubular epithelial cells, and the vascular endothelium ultimately promotes tubulointerstitial fibrosis and irreversible nephron loss [89].

Activation of innate immune signaling pathways appears to be an early event following renal mass reduction. Hemodynamic and metabolic disturbances associated with nephron loss promote the release of damage-associated molecular patterns (DAMPs), which activate Toll-like receptors (TLRs) expressed in resident renal cells. This signaling cascade stimulates the classical NF-κB pathway, resulting in transcription of multiple inflammatory mediators and amplification of the inflammatory response [90,91,92,93]. Consistent with this mechanism, experimental studies have shown increased expression of genes associated with innate immune activation as early as fifteen days after renal ablation in nephrectomized rats [37].

NF-κB signaling plays a central role in this inflammatory cascade. Increased NF-κB activation has been reported in several experimental models of renal injury, including the 5/6 Nx model, where its activation has been detected in the renal interstitium, glomeruli, and vascular compartments [94,95,96,97]. In parallel, activation of the inflammasome pathway, particularly the NLRP3 inflammasome, has been implicated in CKD progression. Experimental evidence demonstrates that NLRP3 activation contributes to renal inflammation and injury in models characterized by severe proteinuria [98]. Activation of this pathway leads to cleavage of caspase-1, which subsequently promotes maturation and release of pro-inflammatory cytokines such as IL-1β and IL-18, thereby amplifying systemic inflammatory signaling [99,100,101].

Elevated circulating levels of inflammatory biomarkers, including C-reactive protein (CRP), interleukin-6 (IL-6), and tumor necrosis factor-α (TNF-α), have been consistently associated with increased cardiovascular risk and mortality [102,103]. Prospective studies have demonstrated that higher CRP levels predict myocardial infarction and other cardiovascular events, even in apparently healthy individuals [102]. Similarly, elevated IL-6 concentrations have been associated with hypoalbuminemia, dyslipidemia, and increased mortality among patients undergoing hemodialysis [103]. These observations support the concept that CKD is a systemic inflammatory disorder with important cardiovascular consequences [104].

Although inflammatory stimuli may be further amplified after initiation of renal replacement therapy due to factors such as vascular access procedures or dialysis membrane biocompatibility [105], that systemic inflammation is already present during earlier stages of CKD. In this regard, Stenvinkel et al. (1999) [106] demonstrated a strong association between inflammation and accelerated atherosclerosis in patients with CKD, highlighting the early onset of systemic inflammatory activation during disease progression.

### 4.2. Oxidative Stress and Redox Imbalance

Oxidative stress is characterized by an imbalance between the excessive production of reactive oxygen species (ROS) and the limited capacity of antioxidant defense systems to neutralize them. In other words, ROS generation exceeds their removal, leading to disruption of redox homeostasis [107]. This oxidative imbalance may induce metabolic alterations and further enhance ROS production, resulting in increased release of superoxide anion (O_2_•^−^), hydroxyl radical (•OH), and hydrogen peroxide (H_2_O_2_). The elevated ROS levels disturb redox balance, consequently reducing antioxidant defenses and culminating in a state of oxidative stress [108].

To counteract the damage caused by excessive ROS, the organism relies on antioxidant systems that are broadly classified into enzymatic and non-enzymatic components, both of which act to attenuate the deleterious effects of pro-oxidant factors. The enzymatic antioxidant system comprises superoxide dismutase (SOD), which catalyzes the dismutation of O_2_•^−^ into H_2_O_2_; catalase (CAT), which converts H_2_O_2_ into water (H_2_O) and molecular oxygen (O_2_); and glutathione S-transferase (GST), which participates in the detoxification of xenobiotics that may or may not induce cellular damage [109].

The non-enzymatic antioxidant system consists primarily of glutathione, present in its reduced (GSH) and oxidized (GSSG) forms. GSH is a tripeptide composed of glutamate, cysteine, and glycine, and plays a central role in maintaining cellular metabolism and redox balance. Deficiency of this compound compromises homeostasis and increases susceptibility to oxidative stress [107].

NADPH oxidase, a major ROS-producing enzyme in renal and vascular tissues, has been strongly associated with the progression of CKD. Experimental evidence demonstrates that two weeks after 5/6 Nx in rats, increased NADPH oxidase activity is already detectable in the aorta and kidneys [87]. Moreover, Shuang Chu et al., 2016 [110] upon analyzing the activity and expression of NADPH oxidase in thoracic aortic tissue from rats 13 weeks after 5/6 Nx, reported significant increases in both enzyme activity and pro-oxidant enzyme expression. In particular, NOX2-derived superoxide has been implicated in vascular oxidative stress and impaired NO signaling, thereby aggravating endothelial dysfunction and increasing vascular resistance in CKD [32]. In contrast, NOX4, one of the most abundant NADPH oxidase isoforms in renal tissue, primarily produces hydrogen peroxide and regulates redox-sensitive signaling pathways involved in cellular adaptation to injury [111]. In humans, a study involving patients with CKD reported that NADPH oxidase and xanthine oxidase generate microvascular ROS, contributing to vascular impairment and endothelial dysfunction [112].

Recent studies employing pharmacological inhibition and ex vivo vascular approaches have provided important insights into the distinct roles of NADPH oxidase (NOX) isoforms in renal redox signaling. Among these, NOX2 and NOX4 emerge as major sources of ROS in the kidney and vasculature; however, their functional impact is highly dependent on cellular localization and the surrounding redox environment [113]. NOX2 is predominantly expressed in inflammatory and vascular cells and is primarily associated with the generation of O_2_•^−^, thereby promoting oxidative stress, endothelial dysfunction, and pro-inflammatory signaling. In contrast, NOX4 is highly expressed in renal tubular epithelial cells, endothelial cells, and vascular smooth muscle cells, where it predominantly generates H_2_O_2_. Due to its constitutive activity and subcellular distribution, NOX4-derived H_2_O_2_ may exert dual effects. At low to moderate concentrations, H_2_O_2_ functions as a signaling molecule involved in the regulation of vascular tone, endothelial function, and cellular homeostasis [114]. Consistently, Muñoz et al. (2018) [115], using isolated intrarenal arteries from male Wistar rats and human renal tissues obtained from nephrectomy specimens of renal tumor patients, demonstrated through pharmacological inhibition of NOX isoforms that NOX2- and NOX4-derived H_2_O_2_ contributes to endothelium-dependent vasodilation, indicating a functional role for NOX-derived ROS beyond oxidative damage.

More recent investigations reinforce the concept that NADPH oxidase-derived ROS constitute a central mechanistic link between renal injury and cardiovascular complications in CKD [32,116,117]. Increased expression of NOX2 and NOX4 has been associated with enhanced oxidative stress and activation of pro-fibrotic pathways, particularly transforming growth factor-β (TGF-β) signaling, promoting extracellular matrix (ECM) accumulation and progressive renal and vascular remodeling [117]. NADPH oxidase-derived ROS are linked to fibrosis and vascular dysfunction [117]. Importantly, the balance between NOX4-derived ROS production and antioxidant defense systems represents a key determinant of whether redox signaling results in adaptive or pathological outcomes.

Serum reduced SOD activity has also been found, whereas malondialdehyde (MDA) levels were elevated in the Nx 5/6 group, 13 weeks after surgery, reflecting enhanced oxidative damage [113]. Another study identified increased renal levels of MDA and thiobarbituric acid reactive substances (TBARSs) in C57BL/6 mice 12 weeks after 5/6 Nx [118]. Furthermore, analysis of mesenteric arteries from rats 12 weeks post-surgery revealed the involvement of ROS in myogenic reactivity [119].

Mitochondrial reactive oxygen species (mtROS) have emerged as key mediators of oxidative stress and cellular injury in CKD [120]. In the 5/6 Nx model, renal mass reduction induces early mitochondrial dysfunction, characterized by impaired oxidative phosphorylation, reduced ATP production, and increased mtROS generation [121]. The accumulation of dysfunctional mitochondria in renal tissue, leading to altered energy metabolism and activation of pro-inflammatory and pro-apoptotic signaling pathways that contribute to disease progression [122]. In addition, time-course analyses indicate that mitochondrial bioenergetic impairment, particularly reduced β-oxidation and respiratory chain activity, persists throughout CKD development, sustaining redox imbalance and ROS overproduction [123]. Importantly, mtROS interacts with other sources of oxidative stress, such as NADPH oxidases, establishing a feed-forward amplification loop that exacerbates endothelial dysfunction, inflammation, and fibrotic remodeling [124]. Consistently, pharmacological targeting of mitochondrial oxidative stress, including the use of mitochondria-directed antioxidants such as Mito-TEMPO, attenuates ROS generation, oxidative damage, and renal fibrosis in 5/6 Nx models, highlighting mtROS as a relevant therapeutic target in CKD [125].

Under physiological conditions, the erythroid 2-related factor 2 (Nrf2) pathway regulates the expression of antioxidant and cytoprotective genes. More broadly, impaired Nrf2 activation has been reported in CKD in both experimental and clinical settings, where it is associated with sustained oxidative stress, inflammation, and tissue injury [126,127,128]. In the 5/6 Nx model, downregulation of the Nrf2-Keap1 pathway exacerbates redox imbalance and limits the adaptive antioxidant response, as evidenced by the lack of improvement in renal function markers despite antioxidant interventions when Nrf2 signaling is insufficiently activated [129]. Conversely, pharmacological activation of Nrf2 has shown renoprotective and vasculoprotective effects. For instance, curcumin-induced Nrf2 nuclear translocation attenuates glomerular hypertension, hyperfiltration, oxidative stress, and the depletion of antioxidant enzymes in 5/6 Nx rats, while also reducing inflammation and fibrosis through modulation of the Nrf2-Keap1 axis [130,131]. Moreover, Nrf2 activation has been implicated in the inhibition of vascular calcification and the preservation of vascular integrity in CKD, highlighting its broader role in cardiorenal protection [132].

### 4.3. Vascular Structural Remodeling

Studies have demonstrated that 5/6 Nx induces significant structural alterations in the vasculature, although the magnitude and characteristics of these changes depend on the experimental context and animal strain. Hamzaoui et al. (2020) [33] reported that, after 12 weeks of 5/6 Nx, mesenteric arteries from C57BL/6JRj mice exhibited an increased baseline external diameter, whereas this change was not observed in 129/Sv mice. However, vascular wall thickening occurred consistently in both strains, suggesting that renal mass reduction promotes vascular hypertrophy across different genetic backgrounds, albeit with strain-specific remodeling patterns [33].

Among the vascular complications associated with CKD, vascular calcification is a particularly prevalent and clinically relevant pathological process. It is defined by the deposition of calcium–phosphate complexes within the vascular wall [133]. This process compromises arterial compliance, leading to increased vascular stiffness and a higher risk of cardiovascular events. Accordingly, patients with CKD exhibit markedly elevated cardiovascular mortality compared with the general population, largely attributable to their increased susceptibility to vascular calcification [134].

Ferrari et al. (2014) [135] showed that rats subjected to 5/6 Nx for 9 weeks exhibited increased calcium content in the abdominal aorta, despite negative von Kossa staining for mineral deposition. These findings suggest that vascular remodeling and biochemical alterations in calcium handling may precede histologically detectable mineralization. More recent evidence indicates that the combination of 5/6 Nx with additional metabolic stressors, such as a high-phosphate, low-protein (HPi-LP) diet, induces medial arterial and cutaneous arteriolar calcification, along with glomerular injury and renal fibrosis [136]. These observations reinforce the concept that 5/6 Nx initiates vascular remodeling, whereas overt calcification depends on model duration and the presence of aggravating factors, particularly disturbances in phosphate–calcium homeostasis.

Although the precise mechanisms underlying vascular calcification remain incompletely understood, the process appears to involve phenotypic transdifferentiation of vascular smooth muscle cells toward an osteogenic profile, accompanied by ECM remodeling. Increased arterial stiffness is associated with the accumulation of collagen-rich ECM, particularly type I and type III collagens, within the vascular wall [137,138]. Consistent with these findings, Ref. [99] demonstrated, using quantitative PCR, increased expression of collagen type I α1 and collagen type III α1 in the thoracic aortas of 5/6 Nx mice.

Arterial stiffness is a key biomechanical alteration linking renal injury to cardiovascular complications in CKD induced by renal mass reduction. The 5/6 Nx model promotes progressive stiffening of large arteries, particularly the aorta, leading to increased systolic blood pressure, reduced vascular compliance, and elevated cardiac afterload [139,140]. This process is driven by ECM remodeling, including increased collagen deposition, elastin disorganization, and enhanced collagen cross-linking [141]. Lysyl oxidase (LOX) plays a central role in this mechanism by stabilizing collagen fibers and promoting arterial wall stiffening. Accordingly, increased LOX expression and activity have been associated with aortic stiffness in 5/6 Nx model, whereas pharmacological inhibition of LOX attenuates collagen cross-linking and improves vascular compliance, identifying this enzyme as a potential therapeutic target [141]. These findings are consistent with earlier evidence demonstrating that CKD induces profound alterations in vascular structure and biomechanics, contributing to increased cardiovascular risk [142].

### 4.4. Endothelial Dysfunction

Endothelial dysfunction is a key mechanism contributing to the progression of CKD and represents an important determinant of the elevated cardiovascular risk observed in affected patients. A central feature of this process is the reduction in NO bioavailability. NO, synthesized from L-arginine by nitric oxide synthase (NOS) enzymes in endothelial cells, plays a critical role in the regulation of vascular tone, platelet aggregation, and inflammatory signaling [26,143].

Experimental studies using the 5/6 Nx model have consistently demonstrated impaired endothelium-dependent vasodilation, particularly in response to acetylcholine (ACh), reflecting endothelial dysfunction similar to that observed in patients with CKD (Table 1) [33,116]. This impairment is primarily associated with reduced eNOS expression or activity, leading to decreased NO production and increased vascular oxidative stress. Multiple molecular mechanisms contribute to this process, including reduced expression of dimethylarginine dimethylaminohydrolase-1 (DDAH1), accumulation of endogenous NOS inhibitors such as asymmetric and symmetric dimethylarginine (ADMA and SDMA), alterations in Akt and glycogen synthase kinase-3 (GSK-3) signaling pathways, and increased activation of NADPH oxidase [144,145,146]. Additional factors further impair NO signaling, including reduced L-arginine transport due to downregulation of the cationic amino acid transporter-1 (CAT-1), elevated circulating levels of trimethylamine-N-oxide (TMAO), and eNOS uncoupling resulting from tetrahydrobiopterin (BH_4_) deficiency [147,148,149].

Evidence from earlier studies further supports the central role of NO signaling in the vascular alterations associated with renal mass reduction. Vaziri et al. (2002) [150] demonstrated that Sprague–Dawley rats subjected to 5/6 Nx exhibit downregulation of eNOS and iNOS expression in the aorta, accompanied by increased lipid peroxidation and oxidative stress, which further contributes to NO inactivation and vascular dysfunction.

Oxidative stress represents another major contributor to endothelial dysfunction in this model. Increased production of ROS, largely mediated by NADPH oxidase activation and mitochondrial dysfunction, promotes NO scavenging and amplifies vascular redox imbalance [19,84]. In addition, oxidation of BH_4_ induces eNOS uncoupling, shifting the enzyme from NO production toward superoxide generation, thereby further aggravating endothelial dysfunction and vascular dysregulation [151].

Activation of the endothelin system also contributes significantly to vascular alterations in the 5/6 Nx model. ET-1 is markedly upregulated in plasma, renal tissue, and vascular beds following renal mass reduction [139]. Enhanced ET-1 signaling through ETA receptors promotes sustained vasoconstriction, vascular smooth muscle cell proliferation, medial hypertrophy, and ECM deposition, leading to structural remodeling of the arterial wall [56]. Moreover, ET-1 signaling exacerbates endothelial dysfunction by reducing NO bioavailability and stimulating oxidative and inflammatory pathways, reinforcing the imbalance between vasodilatory and vasoconstrictor mechanisms [33,151].

Collectively, impaired NO signaling, oxidative stress-induced eNOS uncoupling, and enhanced endothelin activity converge to promote endothelial dysfunction and pathological vascular remodeling in the 5/6 Nx model. These alterations contribute not only to the development and maintenance of hypertension but also to the progression of cardiorenal injury. Consequently, therapeutic strategies aimed at restoring NO bioavailability, reducing oxidative stress, or inhibiting endothelin receptor signaling have demonstrated significant vascular protection in experimental studies, highlighting endothelial dysfunction as a critical therapeutic target in CKD [84,139].

These findings support endothelial dysfunction as a mechanistically relevant vascular endpoint for testing pathway-oriented interventions in the 5/6 Nx model.

**Table 1:** Altered vasoactive pathways in the 5/6 Nx model.

Findings	Strain/Species	Organ	Time After Surgery	References
↓ of protein kinase B/Akt and GSK-3 signaling, and eNOS expression	Male Wistar rat	Thoracic aorta	10 days	[145]
↓ NO bioavailability ↑ NADPH oxidase-derived ROS ↓ eNOS phosphorylation and ↑ eNOS uncoupling	Male Wistar rat	Thoracic aorta	2 weeks	[84]
Inhibition of eNOS, mediated by ↑ ADMA ↓ in the expression of DDAH1	Male Sprague-Dawley rat	Mesenteric artery	5 weeks	[144]
Negative modulation of the CAT-1, reducing arginine transport	Male Wistar rat	Thoracic and abdominal aorta	6 weeks	[147]
↑ of immunoreactive endothelin-1 in both organs	Male Sprague-Dawley rat	Mesenteric arterial bed and thoracic aorta	7 weeks	[58]
↓ of nitric oxide ↑ in ROS and ADMA and SDMA -Inhibition and uncoupling of eNOS	Male Sprague-Dawley rat	Thoracic aorta	8 weeks	[148]
↑ circulating levels of TMAO ↓ of phosphorylated eNOS	Male Sprague-Dawley rat	Thoracic aorta	8 weeks	[149]
On the normal-salt diet, the maximal responses to ACh were unaffected by 5/6 Nx ↓ of ACh dilation in the mesenteric artery of rats subjected to a high-salt diet	Male Sprague-Dawley rat	Mesenteric artery	8 weeks	[39]
↑ expression of AT_1_ receptor, VEGF and α-SMA, and aortic wall thickness, irregularity in the structure of the elastic fibrils in the tunica media, and deterioration in the formation of elastic lamellae	Male Wistar rat	Abdominal aorta	8 weeks	[53]
↓ of ACh vasodilation and phosphorylated eNOS	Male Sprague-Dawley rat	Thoracic aorta	8 weeks	[146]
↓ of ACh vasodilation in the absence and presence of K^+^ channel blockers	Male Sprague-Dawley rat	Intact second- and third-order branches of the mesenteric arteries	10 weeks	[152]
↑ of endothelin-1 in both homozygous and wild-type rats	Homozygous (sl/sl) and wild-type (+/+) rat, dopamine β-hydroxylase-ET_B_ transgenic	Thoracic aorta	12 weeks	[57]
Right and left ventricular myocardial hypertrophy Induced pulmonary vasoconstriction Downregulation of ACE2	Male Sprague-Dawley rat	Heart Pulmonary artery	8 and 14 weeks 14 weeks	[51]
↓ of relaxation to ACh in C57BL/6JRj mice, without change in the relaxation response to NPS ↑ basal external diameter of the mesenteric artery in C57BL/6JRj mice, but not in 129/Sv mice, and an increase in wall thickness in both strains	Male C57BL/6JRj and 129/Sv mice	Segment of first order of mesenteric artery	12 weeks	[33]
↑ of p-IRE1α, GRP78, and mRNA expression levels of the pro-inflammatory cytokines (TNF-α and IL-6) and chemokines (MCP-1 and CX3CL1)	Male homozygous apoE−/− deficient mice	Aorta	12 weeks	[153]
Marked cardiac (total, right and left ventricular) hypertrophy ↑ both left ventricular ACE and ACE2 activity	Male Sprague-Dawley rat	Heart	12 weeks	[52]
↓ of phosphorylated eNOS ↑ of NADPH oxidase and ROS generation	Male Wistar rat	Thoracic aorta	20 weeks	[110]

An increase (↑); a decrease (↓); 5/6 nephrectomy (5/6 Nx); smooth muscle actin alpha (α-SMA); angiotensin-converting enzyme (ACE); acetylcholine (ACh); asymmetric dimethylarginine (ADMA); cationic amino acid transporter-1 (CAT-1); chemokine (C-X3-C motif) ligand 1 (CX3CL1); dimethylarginine dimethylaminohydrolase 1 (DDAH1); endothelial nitric oxide synthase (eNOS); common endoplasmic reticulum stress marker (GRP78); glycogen synthase kinase-3 (GSK-3); interleukin-6 (IL-6); monocyte chemoattractant protein-1 (MCP-1); nicotinamide adenine dinucleotide phosphate (NADPH); sodium nitroprusside (NPS); phosphorylated inositol-requiring enzyme 1α (p-IRE1α); reactive oxygen species (ROS); symmetric dimethylarginine (SDMA); trimethylamine oxide (TMAO); tumor necrosis factor-α (TNF-α); vascular endothelial growth factor (VEGF).

## 5. Temporal Progression of Cardiorenal Alterations in the 5/6 Nx

The progression of cardiorenal alterations following 5/6 Nx occurs in a time-dependent and well-characterized sequence involving progressive interactions between hemodynamic, inflammatory, redox, and vasoactive pathways. After the abrupt loss of nephron mass, compensatory and maladaptive responses develop progressively, including activation of major vasoconstrictor systems such as the RAAS, endothelin signaling, the SNS, and cytochrome P450-derived eicosanoids. In parallel, impairment of vasodilatory pathways, including NO and natriuretic peptide signaling, promotes endothelial dysfunction and vascular remodeling. These mechanisms ultimately contribute to renal injury, systemic hypertension, and cardiovascular complications characteristic of CKD [19,23]. The main stages of this progression and their associated molecular and functional changes are summarized in Table 2.

### 5.1. Early Phase: Acute Renal Mass Reduction (Days—2 Weeks)

Immediately after subtotal Nx, the abrupt reduction in functional nephron number produces marked disturbances in renal hemodynamics [23]. Decreases in renal plasma flow and glomerular filtration rate (GFR) occur within the first days following surgery, accompanied by early tubular injury and the elevation of sensitive biomarkers such as neutrophil gelatinase-associated lipocalin (NGAL) and kidney injury molecule-1 (KIM-1) [154].

At the systemic level, early increases in arterial blood pressure and impairment of endothelium-dependent vasodilation have been reported during this phase (Table 1). These hemodynamic alterations are associated with rapid activation of neurohormonal pathways, including the RAAS and sympathetic nervous system (SNS), which contribute to the initial rise in vascular resistance and renal perfusion instability [84].

Molecularly, early inflammatory activation is also detectable. Experimental studies have demonstrated activation of NF-κB signaling and upregulation of pro-inflammatory cytokines shortly after renal mass reduction [32]. In parallel, redox imbalance emerges as a key early event, characterized by increased superoxide production and alterations in NOS signaling [33,84]. These oxidative bursts contribute to endothelial dysfunction and further aggravate the decline in renal function. Structurally, early glomerular hypertrophy and mild proteinuria [16] may already be observed during this stage (Table 1).

### 5.2. Subacute Phase: Compensatory Hyperfiltration and Oxidative Activation (2–8 Weeks)

During the following weeks after nephron loss, surviving nephrons undergo compensatory hyperfiltration in an attempt to maintain overall filtration capacity. This adaptive response is characterized by increased single-nephron GFR and intraglomerular hypertension. Although these mechanisms transiently preserve renal function, they simultaneously initiate maladaptive processes that promote progressive renal injury [23,44].

Vascular dysfunction becomes more evident during this stage. Reduced NO bioavailability and enhanced responsiveness to vasoconstrictor stimuli reflect progressive endothelial impairment [84,144]. Increased activity of RAAS and endothelin signaling further augments vasoconstrictor tone [56,155], while sympathetic activation contributes to sustained increases in vascular resistance [63]. In addition, alterations in arachidonic acid metabolism contribute to vascular dysfunction. Upregulation of cytochrome P450 enzymes in renal and vascular tissues increases the production of 20-HETE, a potent vasoconstrictor that amplifies the vascular effects of Ang II and endothelin-1 [72].

Inflammatory processes intensify as well. Macrophage infiltration into renal tissue becomes detectable, accompanied by increased expression of profibrotic mediators such as transforming growth factor-β (TGF-β) [16]. In parallel, sustained reactive oxygen species (ROS) production and persistent dysregulation of NOS activity contribute to the maintenance of oxidative stress [32,84,144]. From a structural perspective, progressive albuminuria and proteinuria become more evident, reflecting increasing glomerular barrier dysfunction. Mesangial expansion and early ECM deposition begin to develop during this stage, marking the transition from functional adaptation toward structural renal injury [40,51].

### 5.3. Intermediate Phase: Vascular Dysfunction and Inflammatory Activation (8–12 Weeks)

As disease progression continues, structural renal damage becomes firmly established. Progressive glomerulosclerosis, tubulointerstitial fibrosis, and capillary rarefaction characterize the intermediate stage of the model [16]. During this phase, vascular remodeling becomes increasingly pronounced. Resistance arteries exhibit impaired endothelium-dependent vasodilation, vascular wall thickening, and an increased media-to-lumen ratio, indicating structural adaptation to chronic hemodynamic stress [33,139]. Persistent activation of vasoconstrictor pathways, including RAAS, endothelin signaling, SNS activity, and 20-HETE production, contributes to sustained systemic hypertension. At the same time, vasodilatory systems such as NO and natriuretic peptides become progressively impaired. Reduced NO bioavailability and increased oxidative stress promote endothelial dysfunction and reinforce the imbalance between vasodilatory and vasoconstrictor mechanisms [19,116].

Inflammatory activity remains persistent but evolves into a chronic low-grade inflammatory state characterized by continuous activation of profibrotic signaling pathways [33]. Oxidative stress also persists during this phase, with endothelial redox imbalance contributing to the maintenance of vascular dysfunction [33]. Functionally, animals display clear signs of progressive renal impairment, including reductions in creatinine clearance, elevations in blood urea nitrogen, and sustained proteinuria [33]. Together, these alterations reflect the transition toward a stable phenotype resembling advanced CKD.

### 5.4. Chronic Phase: Fibrosis and Cardiovascular Remodeling (>12 Weeks)

In the chronic stage of 5/6 Nx, renal injury stabilizes into a phenotype consistent with established CKD. Persistent reductions in GFR and sustained proteinuria reflect irreversible nephron loss and progressive fibrotic remodeling of the kidney [16,33]. At the vascular level, endothelial dysfunction becomes pronounced and sustained. Reduced NO bioavailability, persistent oxidative stress, and chronic activation of vasoconstrictor pathways contribute to increased arterial stiffness and sustained systemic hypertension [19].

Cardiac remodeling also develops in parallel with renal disease progression. Experimental studies report left ventricular hypertrophy, myocardial fibrosis, and both systolic and diastolic dysfunction in animals subjected to long-term renal mass reduction dysfunction [18,156]. Under pro-calcifying conditions, particularly when the model is combined with high-phosphate diets, additional cardiovascular abnormalities may arise. These include vascular and valvular calcification, increased arterial stiffness, and mineral deposition within the aortic wall. Such alterations closely recapitulate the cardiovascular complications observed in advanced human CKD [30,40]. These alterations closely resemble the cardiovascular complications observed in advanced human CKD.

This well-defined temporal evolution reinforces the value of the 5/6 Nx model for studying disease progression and testing time-dependent therapeutic interventions.

## 6. Therapeutic Targets in the 5/6 Nx Model: Mechanistic and Translational Relevance

The translational value of the 5/6 Nx model depends on its ability to reproduce pathways targeted by pharmacological interventions. Therefore, therapeutic strategies should be interpreted in the context of their mechanistic effects within this model.

Recent advances in the understanding of CKD pathophysiology have identified several novel therapeutic targets, particularly those related to redox signaling, neurohumoral activation, and regulated cell death pathways. These strategies aim to restore vascular homeostasis and limit progressive renal injury.

As stated above, activation of SNS is a key neurohumoral mechanism that contributes to CKD progression and is consistently reproduced in the 5/6 Nx model. Reduction in renal mass enhances afferent sympathetic signaling to central autonomic nuclei, which leads to SNS overactivity [62,63].

Catheter-based renal denervation (RDN) is, thus, a promising strategy to lower blood pressure by reducing sympathetic activity through renal nerve ablation and causes substantial and sustained blood-pressure reduction, without serious adverse events, in patients with resistant hypertension [157,158,159].

Moreover, it also seems to be effective and safe for CKD hypertensive patients. The results from the Global SYMPLICITY Registry with follow-up data of 3 years showed that RDN is an effective antihypertensive treatment option in CKD patients [160] and the ISAR-denerve study, a randomized study with patients after renal transplantation, has observed that RDN is feasible and safe for these patients [161].

A meta-analysis performed by Xia et al. (2021) [162] showed that RDN may be effective and safe for treating CKD patients with hypertension. Moreover, RDN was associated with reduced albumin excretion during this short follow-up period, and no major complications occurred [162]. Well-designed randomized controlled trials of RDN are urgently needed to confirm the safety and reproducibility of RDN and to assess its impact on clinical outcomes [162].

However, whether RDN is nephroprotective is not fully established. Ott et al. (2015), [163] in an observational pilot study in patients with CKD stages 3 and 4 indicates that treatment of hypertension with RDN decreases BP and slows or even halts the decline of renal function. Kiuchi and Chen (2016) [164] have shown that RDN was able to increase the estimated glomerular filtration, and this effect was more pronounced in CKD patients with uncontrolled hypertension than in controlled hypertension. Hering et al. (2017) [165] observed that RDN can slow further deterioration of renal function irrespective of BP lowering effects in CKD. RDN-induced inhibition of sympathetic outflow to the renal vascular bed may account for improved eGFR via alterations of intrarenal and glomerular hemodynamics.

Moreover, the study of Kiuchi et al. (2016) [166] indicates that renal sympathetic denervation in patients with resistant hypertension and CKD provided both a significant reduction in BP and a substantial reduction in the number of antihypertensive drugs. More importantly, RSD was associated with a long-term increase in eGFR and decrease in albumin excretion, thus curing patients with early-stage CKD.

Therefore, although RDN is a promising strategy to prevent or reverse the renal damage in CKD, more studies are needed to further investigate its benefits in non-hypertensive patients.

Kidney fibrosis correlates well with kidney function and is a therapeutic target in CKD. Since reversal of fibrosis would not lead to regrowth of lost nephrons, lost kidney function would likely not return; so, the primary goal would be to slow down or arrest fibrosis, and thereby prevent further nephron loss and subsequent kidney functional decline [167].

Although SGLT-2 inhibitors, novel mineralocorticoid antagonists, angiotensin-converting enzyme inhibitors and angiotensin 2 receptor antagonists induce antifibrotic effect, none of these are antifibrotic drugs [168].

An overwhelming number of mediators have been implicated in fibrosis, regulating myofibroblast activation, metabolism, inflammation, and ECM cross-linking [169]. TGF-βs are the key cytokines in most fibrosis, so targeting the TGF-b signaling pathway is important. Pirfenidone, which interfere with TGF-b and is approved by the US Food and Drug Administration (FDA) in the treatment of idiopathic pulmonary fibrosis (IPF), seems to present beneficial effects on GFR in the short term (1 year) (NCT00063583, NCT000019590) [170,171]. A Phase 2 trial is ongoing to assess the efficacy of pirfenidone in preventing CKD progression (NCT04258397) [170,172].

Clinical trials using TGF-β-neutralizing monoclonal antibodies (Fresolimumab, LY2382770) did not show any benefit in patients with diabetic kidney disease or Focal Segmental Glomerulosclerosis (NCT01665391) [171,173].

Apoptosis signal-regulating kinase 1 (ASK1) is an upstream signaling kinase of p38 MAPK and JNK in kidney diseases [174]. The phase 2 clinical trial to study the effects of selonsertib, an ASK1 selective inhibitor, in patients with diabetic kidney disease has not met the primary endpoint of change in GFR, although exploratory post hoc analyses suggest that selonsertib may slow diabetic kidney disease progression [175].

A subsequent clinical trial evaluating the safety and efficacy of selonsertib in moderate to advanced DKD (MOSAIC, NCT04026165) demonstrated a slower eGFR decline compared to placebo but also raised potential safety concerns for acute kidney injury [176]. Phase 2 studies evaluating GCS-100 for CKD have no results available.

The usefulness of antifibrotic therapies to prevent or reverse renal fibrosis in humans remains unclear.

According to The Nomenclature Committee on Cell Death (NCCD), regulated cell death (RCD) is a form of cell death that results from the activation of one or more signal transduction modules and hence can be pharmacologically or genetically modulated (at least kinetically and to some extent). RCD plays a major role in development, tissue homeostasis, inflammation, immunity, and multiple pathophysiological conditions [177].

Targeting RCD holds great promise for the treatment of several human disorders and considerable efforts are being made to generate RCD modulators for clinical use [177]. Regarding CKD, it is rational to think that their anti-fibrotic and anti-inflammatory effects would be beneficial to prevent renal fibrosis.

Ferrostatin-1, a potent small molecule that blocks lipid peroxidation and inhibits ferroptosis, a form of regulated, oxidative, nonapoptotic cell death, was protective in isolated kidney proximal tubules, a model of kidney dysfunction [178]. It was able to reduce the death of proximal tubular epithelial cells (PTECs) cultured under hypoxic (1% O_2_) conditions, mimicking the CKD microenvironment [179]. However, although a lead molecule, the presence of a labile ester moiety results in the rapid hydrolysis of Fer-1 into its inactive carboxylic acid, conferring poor metabolic stability [180], and limiting its clinical use.

MCC950, a NLRP3 inflammasome inhibitor, attenuates crystal-induced kidney fibrosis in mice [181], but in a Phase II clinical trial for rheumatoid arthritis, MCC950 induced liver toxicity [182].

In in vitro studies, exogenous mesenchymal stem cells derived-EVs (MSC-EVs) have shown therapeutic potential in reducing inflammation, promoting regeneration and inhibiting fibrosis. Extracellular vesicles contain bioactive substances (such as RNA and proteins) that influence intercellular communication and the regulation of cell death in response to kidney injury [183]. However, there are still some challenges, such as secretion efficiency, long-term safety and clinical efficacy [184]. Engineering modified MSC-EVs may further enhance the effectiveness of EV therapy [183]. As far as we know, there are no clinical studies that regulate cell death in patients with CKD.

Overall, the 5/6 Nx model provides a robust platform for evaluating pharmacological interventions targeting vasoactive and redox pathways. However, the absence of comorbid conditions and interspecies variability must be considered when translating these findings to clinical practice.

## 7. Future Directions

Despite major advances in experimental nephrology, the translation of mechanistic findings into effective therapies for CKD remains limited. Current standard treatments, including inhibitors of RAAS, mainly slow disease progression and rarely prevent long-term decline in renal function. Improved integration between mechanistic preclinical models and clinical research is therefore still needed.

Future work should focus on identifying stage-specific and multi-target therapeutic strategies capable of modulating vascular tone, renal hemodynamics, oxidative stress, inflammation, and fibrosis. In this context, the 5/6 Nx model remains particularly valuable because it reproduces the temporal evolution and mechanistic interaction of these pathways.

Early intervention, personalized experimental design that incorporates sex, age, strain, and diet, and closer alignment between preclinical endpoints and clinical trial outcomes may all improve translational success. Combination approaches involving RAAS modulation, endothelin blockade, antioxidative strategies, and antifibrotic therapy are especially promising avenues for future investigation.

## 8. Conclusions

CKD progression is driven by the interaction of vasoactive, inflammatory, and redox mechanisms. The 5/6 Nx model effectively reproduces these processes, including activation of the RAAS, endothelin signaling, and sympathetic pathways, as well as oxidative stress and endothelial dysfunction. Moreover, the 5/6 Nx model has been extensively validated and reproduces major features of human CKD and presents well-defined temporal progression. Therefore, it is a valuable platform for mechanistic and preclinical studies, as well as translational studies. Overall, the integration of vasoactive and redox-targeted therapies represents a promising strategy for improving outcomes in CKD, and the 5/6 Nx model remains a key tool for advancing this field.

## Figures and Tables

**Table 2:** Time-course alterations in renal and cardiovascular parameters in the 5/6 Nx model of CKD.

Phase	Vascular Alterations	Inflammatory Disturbances	Redox Disturbances	Renal Structural/Functional Changes
Early (Days–2 weeks)	↑ Blood pressure; impaired endothelium-dependent vasodilation	Early NF-κB activation; cytokine upregulation	↑ Superoxide production; altered NOS signaling	↓ Creatinine clearance; early proteinuria; glomerular hypertrophy
Subacute (2–8 weeks)	Reduced NO bioavailability; increased vasoconstrictor responsiveness; mesenteric dysfunction	Macrophage infiltration; ↑ TGF-β signaling	Sustained ROS production; persistent NOS dysregulation	Progressive albuminuria; mesangial expansion; ECM deposition
Intermediate (8–12 weeks)	Impaired flow-mediated dilation; vascular remodeling; increased media-to-lumen ratio	Chronic low-grade inflammation; profibrotic signaling	Persistent oxidative stress; endothelial redox imbalance	Established glomerulosclerosis; tubulointerstitial fibrosis
Chronic (>12 weeks)	Sustained endothelial dysfunction; arterial stiffness; systemic hypertension	Stabilized fibrotic pathways; inflammatory persistence	Ongoing oxidative burden; antioxidant system impairment	Reduced GFR; stable CKD phenotype; persistent proteinuria

An increase (↑); a decrease (↓); nuclear factor kappa B (NF-κB); nitric oxide synthase (NOS); transforming growth factor beta (TGF-β); reactive oxygen species (ROS); extracellular matrix (ECM); glomerular filtration rate (GFR); chronic kidney disease (CKD).

## Data Availability

No new data were created or analyzed in this study. Data sharing is not applicable.

## Abbreviations

The following abbreviations are used in this manuscript:

129/Sv	An inbred mouse strain widely used as a source of embryonic stem (ES) cells for genetic targeting.
5/6 Nx	five-sixths nephrectomy
20-HETE	20-hydroxyeicosatetraenoic acid
ACE	angiotensin-converting enzyme
ACE2	angiotensin-converting enzyme 2
ACh	acetylcholine
ADMA	asymmetric dimethylarginine
Akt	protein kinase B
ANP	atrial natriuretic peptide
AT_1_	angiotensin II type 1 receptor
AT_2_	angiotensin II type 2 receptor
BH_4_	tetrahydrobiopterin
BK_Ca_	large-conductance calcium-activated potassium channel
BNP	B-type natriuretic peptide
C57BL/6JRj	An inbred mouse strain originating from the Jackson Laboratory (J) and commercialized by Janvier Labs (Rj).
CAT-1	cationic amino acid transporter-1
cGMP	cyclic guanosine monophosphate
CKD	chronic kidney disease
CYP	cytochrome P450
DAG	diacylglycerol
DAMPs	damage-associated molecular patterns
DDAH1	dimethylarginine dimethylaminohydrolase 1
ECE	endothelin-converting enzyme
ECM	extracellular matrix
eNOS	endothelial nitric oxide synthase
ETA	endothelin receptor type A
ETB	endothelin receptor type B
ET-1	endothelin-1
GFR	glomerular filtration rate
GRP78	78 kDa glucose-regulated protein
GSH	reduced glutathione
GSK-3	glycogen synthase kinase-3
GSSG	oxidized glutathione
GST	glutathione S-transferase
H_2_O	water
H_2_O_2_	hydrogen peroxide
HPi-LP	high-phosphate, low-protein
IL-1β	interleukin-1 beta
IL-6	interleukin-6
IL-18	interleukin-18
iNOS	inducible nitric oxide synthase
IP_3_	inositol 1,4,5-trisphosphate
KIM-1	kidney injury molecule-1
LOX	lysyl oxidase
MCP-1	monocyte chemoattractant protein-1
MDA	malondialdehyde
MLCK	myosin light-chain kinase
mtROS	mitochondrial reactive oxygen species
NADPH	nicotinamide adenine dinucleotide phosphate
NE	norepinephrine
NF-κB	nuclear factor kappa B
NGAL	neutrophil gelatinase-associated lipocalin
NLRP3	NOD-like receptor family pyrin domain containing 3
NO	nitric oxide
NOX	NADPH oxidase
NOX2	NADPH oxidase 2
NOX4	NADPH oxidase 4
NPR-A	natriuretic peptide receptor A
NPR-B	natriuretic peptide receptor B
NPS	sodium nitroprusside
O_2_	oxygen
O_2_^•−^	superoxide anion
OH^•^	hydroxyl radical
PKG	protein kinase G
PLC	phospholipase C
PMCA	plasma membrane Ca^2+^-ATPase
PTEC	proximal tubular epithelial cells
RAAS	renin-angiotensin-aldosterone system
RCD	regulated cell death
RDN	renal denervation
ROS	reactive oxygen species
SDMA	symmetric dimethylarginine
SERCA	sarco/endoplasmic reticulum Ca^2+^-ATPase
SGLT-2	sodium–glucose cotransporter 2
SOD	superoxide dismutase
SR	sarcoplasmic reticulum
TBARS	thiobarbituric acid reactive substances
TGF-β	transforming growth factor beta
TLRs	Toll-like receptors
TMAO	trimethylamine N-oxide
TNF-α	tumor necrosis factor alpha
VEGF	vascular endothelial growth factor
α-SMA	alpha-smooth muscle actin
